# In vitro anti-malarial interaction and gametocytocidal activity of cryptolepine

**DOI:** 10.1186/s12936-017-2142-z

**Published:** 2017-12-28

**Authors:** Arnold Donkor Forkuo, Charles Ansah, Kwesi Boadu Mensah, Kofi Annan, Ben Gyan, Anjo Theron, Dalu Mancama, Colin W. Wright

**Affiliations:** 1 Department of Pharmacology, Faculty of Pharmacy and Pharmaceutical Sciences, College of Health Sciences, Kwame Nkrumah University of Science and Technology, Kumasi, Ghana; 2 Department of Immunology, Noguchi Memorial Institute for Biomedical Research, University of Ghana, Legon, Ghana; 3 Biosciences, Council for Scientific and Industrial Research, P.O. Box 395, Pretoria, 0001 South Africa; 4 School of Pharmacy, University of Bradford, West Yorkshire, BD7 1DP UK

**Keywords:** Gametocytocidal, Malaria, Anti-malarial drug combinations, *Cryptolepis sanguinolenta*, Cryptolepine

## Abstract

**Background:**

Discovery of novel gametocytocidal molecules is a major pharmacological strategy in the elimination and eradication of malaria. The high patronage of the aqueous root extract of the popular West African anti-malarial plant *Cryptolepis sanguinolenta* (Periplocaceae) in traditional and hospital settings in Ghana has directed this study investigating the gametocytocidal activity of the plant and its major alkaloid, cryptolepine. This study also investigates the anti-malarial interaction of cryptolepine with standard anti-malarials, as the search for new anti-malarial combinations continues.

**Methods:**

The resazurin-based assay was employed in evaluating the gametocytocidal properties of *C. sanguinolenta* and cryptolepine against the late stage (IV/V) gametocytes of *Plasmodium falciparum* (NF54). A fixed ratio method based on the SYBR Green I fluorescence-based assay was used to build isobolograms from a combination of cryptolepine with four standard anti-malarial drugs in vitro using the chloroquine sensitive strain 3D7.

**Results:**

*Cryptolepis sanguinolenta* (IC_50_ = 49.65 nM) and its major alkaloid, cryptolepine (IC_50_ = 1965 nM), showed high inhibitory activity against the late stage gametocytes of *P. falciparum* (NF54). In the interaction assays in asexual stage, cryptolepine showed an additive effect with both lumefantrine and chloroquine with mean ΣFIC_50_s of 1.017 ± 0.06 and 1.465 ± 0.17, respectively. Cryptolepine combination with amodiaquine at therapeutically relevant concentration ratios showed a synergistic effect (mean ΣFIC_50_ = 0.287 ± 0.10) whereas an antagonistic activity (mean ΣFIC_50_ = 4.182 ± 0.99) was seen with mefloquine.

**Conclusions:**

The findings of this study shed light on the high gametocytocidal properties of *C. sanguinolenta* and cryptolepine attributing their potent anti-malarial activity mainly to their effect on both the sexual and asexual stages of the parasite. Amodiaquine is a potential drug partner for cryptolepine in the development of novel fixed dose combinations.

## Background

Malaria still remains a disease of public health importance with an estimated 3.3 billion people at risk globally and 214 million new cases with 438,000 deaths reported in 2015. The sub-Saharan region of Africa accounts for an estimated 90% of all deaths due to malaria, with children under 5 years of age accounting for 78% of these [1].

Despite the successes seen in the approach to reduce the number of deaths associated with malaria, this disease still remains a grave public health problem. The majority of the currently used anti-malarial drugs were intended to target the symptom-causing pathogenic blood stages in man and to contend with the continuous risk of drug resistance [2]. Nonetheless, to achieve the global objective of malaria eradication, medicines with activity against parasite transmission [3] and the hepatic stages responsible for disease relapse also need to be developed. The reported development of *Plasmodium falciparum* resistance to the most potent anti-malarial agents (the artemisinins) in the Thai–Cambodia border puts the progress achieved under serious threat [4, 5]. This scenario requires the urgent need to accelerate the discovery and development of novel anti-malarial leads and combinations that have activity against both the sexual and asexual stages of the parasite.

For this reason, the popular West African anti-malarial plant *Cryptolepis sanguinolenta* (Periplocaceae) and it major alkaloid, cryptolepine were assessed against the transmissible stages of the human malaria parasite. The aqueous root extract of *C. sanguinolenta* has been used for decades in West Africa for the treatment of malaria [6] and currently, several herbal preparations registered for use in Ghanaian orthodox clinics contain the aqueous root extract of the plant. The wide usage of the plant may be attributed to the safety and high cure rate of a tea bag formulation (Phyto-laria) of the plant against chloroquine resistant strains of *P. falciparum* in human clinical trials [7], supporting their usage as alternative tools to the standard anti-malarial interventions or as complementary treatments in the absence of standard anti-malarial drugs. Cryptolepine, the major indoloquinoline alkaloid isolated from the plant has the most potent antiplasmodial activity [8] and has been shown to exhibit potent in vitro activities against both chloroquine-sensitive and chloroquine-resistant *P. falciparum* [9].

**Fig. 1:**
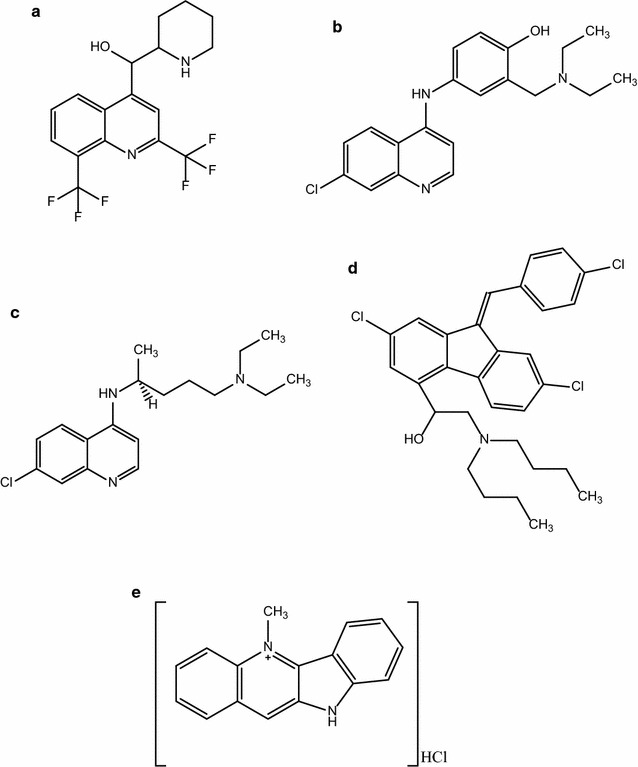
Structure of anti-malarial agents used in the interaction assay. **a** Mefloquine, **b** amodiaquine, **c** chloroquine, **d** lumefantrine, **e** crytolepine hydrochloride

With lessons learnt from the widely used artemisinin derivatives, thus their isolation from the medicinal plant *Artemisia annua*, directed the exploration of the popularly used anti-malarial plant, *C. sanguinolenta* and its major alkaloid, cryptolepine for possible gametocytocidal effects on *P. falciparum*. The in vitro anti-malarial interaction of cryptolepine with chloroquine, lumefantrine, amodiaquine and mefloquine (Fig. 1) were also ascertained in this study.

## Methods

### Plant material

The sun-dried cut roots of *C. sanguinolenta* used in this study were collected at the Centre for Scientific Research into Plant Medicine (CSRPM), Mampong-Akwapim, Ghana in August 2012 and were identified by the Plant Development Centre of the Institution. Its authenticity was confirmed by Dr. Kofi Annan of the Department of Pharmacognosy, KNUST and subsequently compared to a voucher specimen KNUST/HM1/2008/L056 at the herbarium of the Department of Pharmacognosy/Herbal Medicine, College of Health Sciences.

The powdered roots (650 g) were boiled for 30 min with 5 L of distilled water, decanted and filtered. The filtrate was freeze dried to obtain a freeze-dried sample of the crude extract (yield = 11.08% w/w) referred to as cryptolepis (CPS). Cryptolepis was reconstituted in distilled water prior to use in the gametocytocidal assay.

### Drugs/chemicals

Lumefantrine, chloroquine, mefloquine and amodiaquine were purchased from Sigma-Aldrich (St. Louis, MO, USA). Gentamicin was obtained from Invitrogen Life Technologies Inc. (Carlsbad, CA, USA). RPMI-1640 medium, streptomycin/penicillin, l-glutamine and HEPES were obtained from Gibco BRL Life Technologies (Grand Island, NY, USA). Cryptolepine hydrochloride (purity 98.9%) was isolated from the root of *C. sanguinolenta* as described by Kuntworbe et al. [10].

### In vitro cultivation of asexual stage *Plasmodium falciparum*

The in vitro cultivation method of *P. falciparum* drug-sensitive strain NF54 was adapted from Reader et al. [11]. Parasite cultures were maintained in human erythrocytes (5% haematocrit, A rhesus positive) suspended in complete parasite medium (CPM) (RPMI 1640 medium supplemented with 25 mM HEPES, 0.2% d-glucose, 24 μg/mL gentamicin, 0.2% sodium bicarbonate (pH = 7.3), 200 μM hypoxanthine with 10% human serum (A rhesus positive) and flushed with 90% N_2_, 5% O_2_, and 5% CO_2_ in humidified modular chambers at 37 °C. Parasite medium was changed daily and fresh CPM introduced. Parasitaemia of Giemsa-stained slides were monitored daily with light microscopy.

### Induction of gametocytogenesis and maintenance of gametocyte cultures

Combined conditions of low haematocrit and nutrient starvation were employed in the induction of gametocytogenesis. To trigger gametocytogenesis, asexual cultures of 6–10% parasitaemia were diluted to 0.5% parasitaemia at 6% haematocrit and then introduced into a glucose-free medium. The medium was kept at 37 °C gassed with 90% N_2_, 5% O_2_, and 5% CO_2_ without shaking. The medium was changed daily. After 72 h, the haematocrit was dropped to 3% (day 0). Gametocytogenesis was monitored daily before medium (glucose-free) was changed. To eliminate residual asexual parasites, cultures were treated on days 6–9 by continuous treatment with 50 mM *N*-acetyl glucosamine (NAG). The medium was then fortified with 0.2% glucose from day 10 onwards. The gametocyte levels were monitored daily by microscopy until they were predominantly (> 90%) stage V and these gametocytes were employed in the resazurin-based assay.

### Gametocyte viability assays

The in vitro gametocytocidal activities of cryptolepine and the aqueous root extract of *C. sanguinolenta* were measured by assessing gametocyte survival after drug exposure using resazurin-based assay. The resazurin-based assay was based on method described by Tanaka et al. [12]. Eleven drug concentrations of cryptolepine, the aqueous root extract of *C. sanguinolenta* and dihydroartemisinin ranging from 10 µM to 1.69 × 10^−4^ µM (threefold serial dilutions) prepared in distilled water were placed in triplicate in a transparent 96-well flat bottom plates. Parasitized RBCs were added to a final concentration of 5% haematocrit, 2% gametocytaemia in a total incubation volume of 100 µL.

Dihydroartemisinin (10 µM) was used as a reference standard in the drug assay. The drug plate was placed on a mechanical shaker for 20 s before being encased in an air-tight chamber and gassed for 5 min with a 5% O_2_, 5% CO_2_ and a balanced N_2_ mixture. The plates were incubated at 37 °C for 48 h after which 10 µL of resazurin-based assay reagent was added to each well and the plate was shaken for 20 s. The plate was left to incubate for 2 h and then centrifuged at 120×*g* for 1 min. The supernatant (70 µL) was transferred to a clean 96-well plate before being read in a multiwell spectrophotometer (Infinite F500, Tecan, USA) by fluorescence detection at 535/612 nm. Gametocyte survival in each test culture after treatment was calculated relative to the control wells. The experiment was performed in triplicate on two separate occasions.

### Antiplasmodial interaction assay

#### In vitro malaria parasite cultivation

*Plasmodium falciparum* laboratory strain 3D7 was cultured according to method described by Trager and Jensen [13] with slight modifications as described. Human erythrocytes (O rhesus positive) fortified in complete culture medium (pH 7.3) served as host cells for parasite maturation. The complete culture medium consisted of filter-sterilized RPMI 1640 solution supplemented with 0.5% AlbuMAX II, 0.72% HEPES (*N*-2-hydroxyethylpiperazine-*N*-2-ethanesulfonic acid) and hypoxanthine buffered with 0.4% sodium bicarbonate (NaHCO_3_). Gentamicin (0.005 mg/mL) was added to the final solution.

A controlled experimental environment with a gas supply of 92% N_2_, 5% CO_2_ and 3% O_2_ at 37 °C was used to grow the parasites in culture flasks. Parasite growth, viability and stages were monitored daily by light microscopy of Giemsa-stained cultures after changing the culture media. Parasitaemia levels were kept between 2 and 8%, with a 5% haematocrit.

### In vitro antiplasmodial interaction assay

The SYBR Green I-based fluorescence assay was employed to investigate the in vitro anti-malarial interaction between cryptolepine and the four standard anti-malarial agents against *P. falciparum* (chloroquine sensitive, 3D7). Cryptolepine (Drug A, CPE), lumefantrine (LUM), mefloquine (MFQ), chloroquine (CQ) and amodiaquine (AMQ) stock solutions were prepared in ethanol at 1 mM. Concentrations ranging from 32.5 to 2080 nM for CPE, 2 to 640 nM for LUM, 3.2 to 83 nM for CQ, 5 to 800 nM for MFQ and 1 to 128 nM for AMQ were used for the interaction assays. A fixed ratio interaction assay as described previously by Fivelman et al. [14] was employed in this assay.

For each assay, fixed drug ratios (4:1, 3:2, 2:3 and 1:4) were prepared in a 10 µL volume for cryptolepine (Drug A) and any of the standard agents (Drug B). A twofold serial dilution followed each fixed ratio in a well ensuring the IC_50_ of each drug alone (5:0 and 0:5) fell approximately at the mid-point of the serial dilution. Parasite culture (90 µL) was added to each well to obtain seven desired final concentrations for the assay. The final haematocrit and parasitaemia of the culture media were 2 and 1%, respectively. The assay plates were gassed with a mixture of gases containing 92% N_2_, 5% CO_2_ and 3% O_2_ for 6 min and carefully arranged in a modular incubator (Billups-Rothenberg Inc, USA). The plates were incubated at 37 °C for 48 h. Post incubation, the plates were stored at − 30 °C wrapped in aluminium foil. The plates were thawed and subsequently mixed with 100 μL of Malaria SYBR Green 1 fluorescent (MSF) lysis buffer containing SYBR Green. The plates were incubated at room temperature in the dark for an hour and fluorescence data (relative fluorescence unit, RFU) were acquired using a fluorescence multi-well plate reader (Tecan Infinite M200 Pro) with emission and excitation wavelength at 535 and 485 nm, respectively. The experiment was performed in triplicate.

### Statistical analysis

The performance of the gametocyte assay was assessed by estimating the Z factor statistical parameter described by Zhang et al. [15]. Gametocyte viability was calculated using the relation below

$$\% {\text{ Parasite viability}} = \frac{{\left( {Fluorescence_{compound} - Mean \,fluorescence_{background} } \right)}}{{\left( {Mean \,fluorescence_{positive} - Mean \,fluorescence_{background} } \right)}} \times 100$$

The IC_50_ (50% inhibitory concentration) served as a measure of anti-malarial activity and was determined by plotting and analysing dose–response curves using GraphPad Prism (GraphPad 6 Software, San Diego, USA). The assessment of drug interaction was based on calculating the sum of the fractional inhibitory concentration (ΣFIC_50_s) at the given effective concentration by the formula below

$$\sum {\text{FIC}}_{{50}} = \left( {\frac{{IC_{{50}} \;{\text{of}}\;{\text{cryptolepine}}\;{\text{in}}\;{\text{combination}}}}{{IC_{{50}} \;{\text{of}}\;{\text{cryptolepine}}\;{\text{alone}}}}} \right) + \left( {\frac{{IC_{{50}} \;{\text{of}}\;{\text{Drug}}\;{\text{B}}\;{\text{in}}\;{\text{combination}}}}{{IC_{{50}} \;{\text{of}}\;{\text{Drug}}\;{\text{B}}\;{\text{alone}}}}} \right)$$

The nature of interaction was explained using ΣFIC_50_s. Values < 0.8 denote synergism, 0.8–1.4 denotes additive interaction, and ≥ 1.4 denotes antagonism [16]. The overall nature of the anti-malarial interaction was based on the mean ΣFIC_50_s.

A Microsoft Excel datasheet was used to calculate percentage inhibition in relation to the control results. Figures were made using GraphPad Prism for Windows version 6.0 (GraphPad Software, San Diego, CA, USA).

## Results

### Gametocyte viability assay

**Fig. 2:**
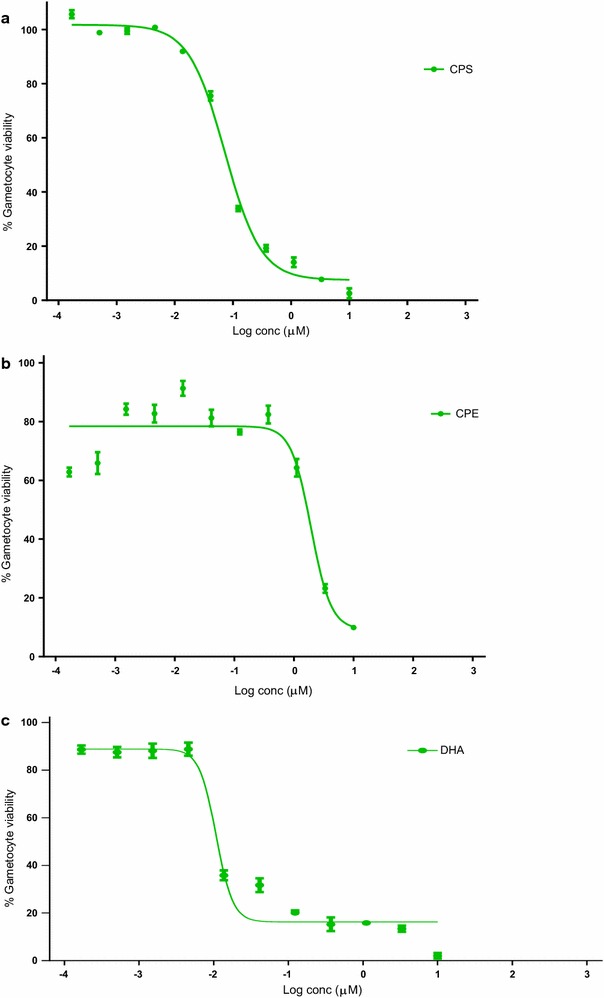
Gametocyte viability of CPS and CPE. Gametocytocidal activity of CPS (**a**), CPE (**b**) and DHA (**c**) against late stage gametocytes. Bars represent mean gametocyte activity at each compound concentration (with standard deviation [SD])

Data from the late stage gametocyte assay indicates a preferential inhibition of the viability of mature sexual-stage parasites by the aqueous extract of *C. sanguinolenta* and its major alkaloid cryptolepine. The reduction in gametocytes was in a dose-dependent fashion in all drug treatments and statistically significant. IC_50_ values of 49.65 and 1965 nM were recorded for CPS and CPE, respectively (Fig. 2). The Z-factor for the assay was calculated to be 0.98, indicating that the assay performed very well. *Cryptolepis sanguinolenta* however, demonstrated more potent activity against the late stage gametocytes compared to its major alkaloid, cryptolepine. The reference standard, dihydroartemisinin (10–1.69 × 10^−4^ µM) exhibited an IC_50_ of 15 nM (Fig. 2) falling within the range normally expected for the resazurin-based assay and other gametocytocidal assay platforms, as described by Reader et al. [11].

Taken together, *C. sanguinolenta* (CPS) and it major alkaloid, cryptolepine (CPE) have potent inhibitory effects on the late stage gametocyte of *P. falciparum* strain NF54 (Fig. 2).

### In vitro antiplasmodial interaction

**Fig. 3:**
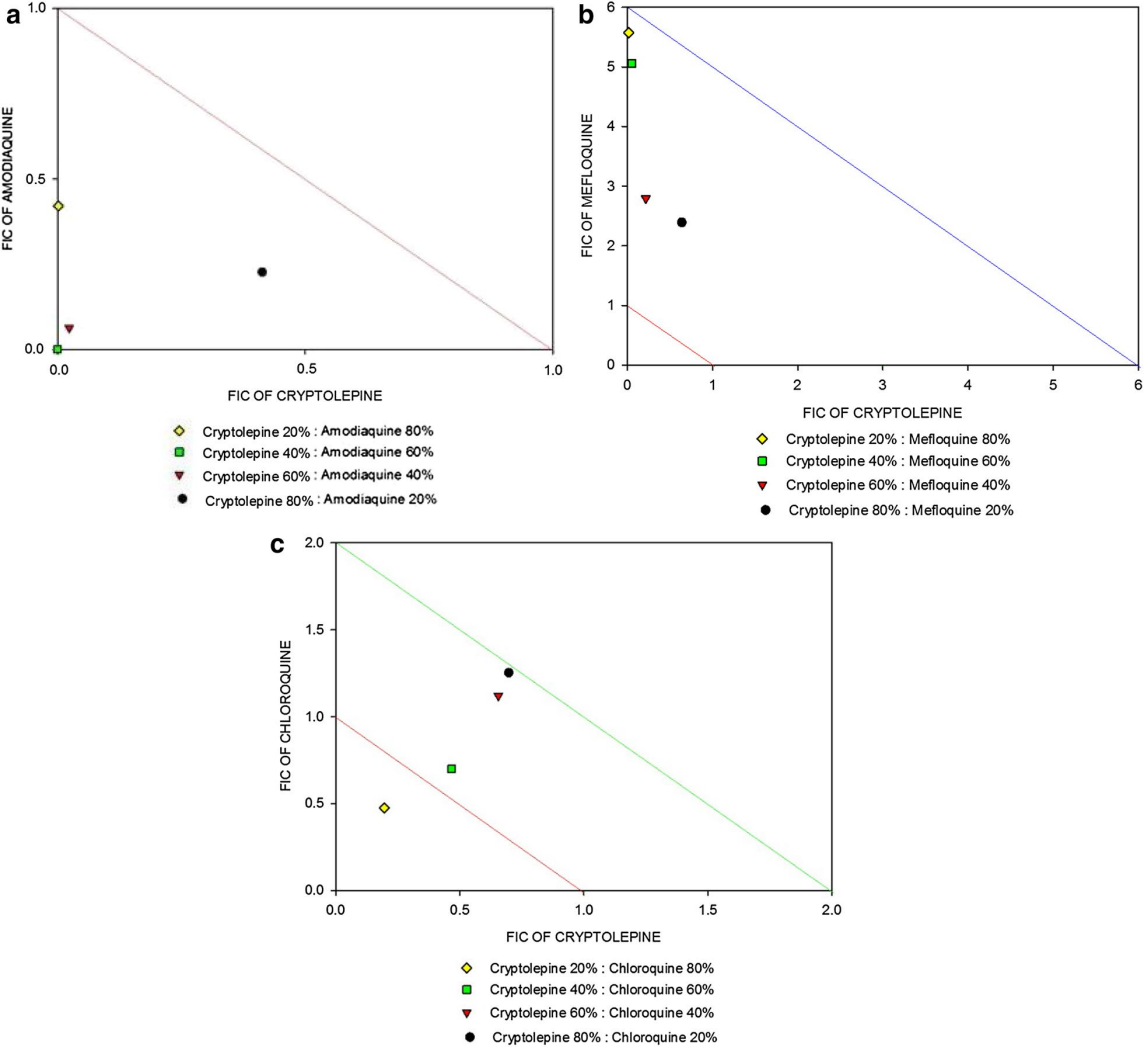
Effects of combinations of cryptolepine with standard anti-malarial drugs on *Plasmodium falciparum* growth in vitro (3D7 strain). Isobolograms illustrating the effect of combinations of cryptolepine with amodiaquine (**a**), mefloquine (**b**), and chloroquine (**c**). The interaction between cryptolepine and amodiaquine, mefloquine, chloroquine or lumefantrine against ring stage parasites was determined using the SYBR Green I fluorescence-based drug sensitivity assay with the fixed ratio method. Each combination was set up in triplicate for 48 h. The FIC_50_ concentrations were used for plotting the isobologram

The in vitro IC_50_s of cryptolepine and the standard anti-malarial drugs in the asexual stages of *P. falciparum* 3D7 are presented in Table 1. In the in vitro interaction assays, cryptolepine’s combination with chloroquine or lumefantrine showed additivity with a mean ΣFIC_50_ of 1.342 ± 0.34 and 1.017 ± 0.45, respectively (Table 2). Cryptolepine combination with amodiaquine at all therapeutically relevant ratios showed a mean ΣFIC_50_ (0.235 ± 0.15) of less than 0.8 suggesting synergism. Antagonism (mean ΣFIC_50_ = 4.182 ± 0.68) was observed when cryptolepine was combined with mefloquine. Isobolograms indicating various cryptolepine-based combinations are presented in Fig. 3.

**Table 1:** In vitro anti-malarial activity against asexual blood stages

Drugs	Mean IC_50_ ± S.E.M (nM)
Amodiaquine	12.63 ± 1.66
Mefloquine	12.98 ± 0.47
Lumefantrine	10.80 ± 0.97
Chloroquine	11.05 ± 1.79
Cryptolepine	603.82 ± 75.57

IC_50_ values of cryptolepine, lumefantrine, mefloquine, and chloroquine against *Plasmodium falciparum* strain 3D7 are expressed as mean ± standard error of mean of the results of at least three independent assays

**Table 2:** In vitro interaction of cryptolepine combinations against 3D7 *P. falciparum* strains

Drug combination	ΣFIC_50_				Mean ΣFIC_50_	Interaction
	4:1	3:2	2:3	1:4		
Cryptolepine + amodiaquine	0.640 ± 0.08	0.0234 ± 0.11	0.0631 ± 0.07	0.421 ± 0.12	0.287 ± 0.10	Synergism
Cryptolepine + mefloquine	3.015 ± 0.91	3.013 ± 1.01	5.106 ± 1.13	5.595 ± 0.91	4.182 ± 0.99	Antagonism
Cryptolepine + lumefantrine	2.1108 ± 0.17	1.6865 ± 0.01	0.1475 ± 0.02	0.1245 ± 0.04	1.017 ± 0.06	Additivity
Cryptolepine + chloroquine	1.95 ± 0.21	1.775 ± 0.36	1.66 ± 0.04	0.475 ± 0.05	1.465 ± 0.17	Additivity

The ratios 4:1, 3:2, 2:3 and 1:4 refer to fixed dosage ratios for drug A (cryptolepine) to drug B (amodiaquine, mefloquine, lumefantrine, chloroquine). Values are the means from ≥ 3 experiments

## Discussion

The aqueous root extract of *C. sanguinolenta* has gained wide usage as an anti-malarial agent in both herbal and orthodox hospitals in Ghana and other West African countries. This coupled with the plant’s established efficacy against the asexual stages of *P. falciparum* [7, 17] motivated this study to evaluate possible gametocytocidal properties of the plant and its major alkaloid, cryptolepine. In the current study, the stage-specific in vitro gametocytocidal activities of *C. sanguinolenta* (CPS) and its major alkaloid cryptolepine (CPE) were tested against the late-stage NF54 strain of the malaria parasite, *P. falciparum.* The assay assesses gametocyte survival after drug exposure using resazurin-based assay, and indicated a potent loss of viability of the late stage gametocyte with both *C. sanguinolenta* (49.65 nM) and cryptolepine (1965 nM). This result is not surprising as earlier work [18] demonstrated potent in vitro loss of viability of the late stage gametocytes by local polyherbal anti-malarial products containing *C. sanguinolenta*. Despite the demonstrated activity, the major alkaloid from the plant known to possess the most efficacious in vitro antiplasmodial activity showed less gametocytocidal activity compared to the root extract of the plant. This indicates the presence of other alkaloids in the aqueous root extract of the plant contributing to the high activity against gametocytes. Given the demonstrated high gametocytocidal properties of CPS and CPE, their prominent antiprotozoal activity may be attributed mainly to their effect on both the asexual and sexual stages of the *Plasmodium* parasite.

Our current study also sheds light on the in vitro anti-malarial interaction of cryptolepine with four standard anti-malarial agents. The steady rise in the resistance of *P. falciparum* to current anti-malarial drugs, has directed current therapeutic strategies toward the development of combination therapies. Cryptolepine is a promising anti-malarial compound effective against both chloroquine-sensitive and chloroquine-resistant *P. falciparum* [9]. It has however been shown to possess DNA intercalating and topoisomerase II inhibitory effect [19, 20]. This has directed research into structural modification of the compound to synthesize relatively safer derivatives. Previous pharmacokinetic reports of cryptolepine showed a rapid disappearance from the plasma and localization in various tissues except the CNS with possible hepatobiliary clearance pathway [21]. Aldehyde oxidase has been shown to be involved in the metabolism of cryptolepine [22]. Nanoformulation of cryptolepine hydrochloride demonstrated a better in vivo antiplasmodial chemosuppression, superior bioavailability and an increased half-life compared with the free compound [23]. A candidate drug partner is sought for the parallel development of cryptolepine, comparable to ACT encouraged by the WHO. This approach will provide cryptolepine with potential drug partners for further development to reduce the risk of drug resistance to *P. falciparum*. A new anti-malarial combination should preferably target both the rapidly replicating asexual stages and the less metabolically active, nonreplicating mature gametocytes.

The in vitro anti-malarial interaction of cryptolepine using the SYBR Green fluorescent assay demonstrated varied interaction with these standard agents. Cryptolepine showed varied interaction with the 4-aminoquinolines, amodiaquine and chloroquine. The combination of cryptolepine with amodiaquine had a synergistic effect in vitro (mean ΣFIC = 0.235 ± 0.15) whereas an additive effect (mean ΣFIC = 1.342 ± 0.34) was seen with chloroquine. The 4-aminoquinolines are considered to share, in principle, the same mode of action. However, a different interactive profile was found with cryptolepine. It is therefore wrong to equate amodiaquine to chloroquine in terms of activity. The synergy observed with amodiaquine may be due to the Mannich base structure in amodiaquine. A similar synergistic effect attributed to the Mannich base side chain in pyronaridine and amodiaquine has been reported with artemisinin [24]. An antagonistic effect was observed when cryptolepine was combined with mefloquine. Mefloquine has been shown to inhibit the uptake of chloroquine as well as chloroquine’s ability to cause the accumulation of undigested haemoglobin [25].

Mefloquine (and possibly quinine) has also been hypothesized to inhibit endocytosis of the erythrocyte cytosol by the parasite, resulting in a lowered free haem concentration, to which chloroquine binds, in the digestive vacuole [25]. Cryptolepine, an indoloquinoline has been shown to act similarly to chloroquine by inhibiting the biomineralization of the toxic waste material haem into an insoluble complex, haemozoin, leading to membrane damage and parasite death [9, 26, 27]. On this background it is not surprising that antagonism was observed when cryptolepine was combined with mefloquine, possibly following a similar pathway of antagonism demonstrated by mefloquine when combined with chloroquine. In the case of cryptolepine-lumefantrine combination at therapeutically relevant concentration ratios, an additivity effect (mean ΣFIC = 1.017 ± 0.45) was observed.

The combination of cryptolepine with amodiaquine showed synergy in vitro in *P. falciparum* strain 3D7 and this combination provides a dual action with both agents inhibiting haemoglobin digestion in the asexual blood stages and amodiaquine inhibiting gametocyte maturation/gamete exflagellation by different mechanisms [28]. Such combinations with dual action are relevant in malaria endemic regions, where infections are usually asymptomatic with clinical symptoms developing late in the course of the disease, permitting the maturation of gametocytes and hence disease transmission [29, 30]. The enhanced anti-malarial activity of cryptolepine with amodiaquine may possibly result in low-dose treatment regimens and hence reduce toxicity. However, it should be noted that these assay results may vary in different plasmodium strains and further investigations is recommended in the early stages of gametocytes and in several *P. falciparum* strains and/or rodent malaria models to accentuate the interactions established.

## Conclusions

*Cryptolepis sanguinolenta* and its major alkaloid, cryptolepine exhibited high activities against the late-stage gametocyte of *P. falciparum* NF54, attributing their primary targets as the mature gametocytes and intraerythrocytic parasite. Lumefantrine and chloroquine both showed additivity when each drug was combined with cryptolepine whereas antagonism was observed when cryptolepine was combined with mefloquine at therapeutically relevant concentration ratios. Cryptolepine in combination with the Mannich base, amodiaquine, showed synergism. On this basis, the cryptolepine–amodiaquine combination certainly deserves attention for further studies in regards to the synergy demonstrated.

## Abbreviations

FIC: fractional inhibitory concentrations
Mean ΣFICs: mean sums of fractional inhibitory concentrations
IC50: 50% inhibitory concentration
WHO: World Health Organization
ACT: artemisinin-based combination therapy
CPE: cryptolepine
CPS: aqueous root extract of *Cryptolepis sanguinolenta*
LUM: lumefantrine
AMQ: amodiaquine
CQ: chloroquine
nM: nano-molar
mM: milli-molar
CPM: complete parasite medium
RBC: red blood cell
MSF: malaria SYBR Green I fluorescence
